# Single blood drop diagnostic activation test (DAT) for food allergies

**DOI:** 10.1186/2045-7022-1-S1-P75

**Published:** 2011-08-12

**Authors:** Amit Singht, Scott Seki, Marco Garcia, Kevin Mckee, Trevor Longbottom, Brittany Weldon, Grace Yu, Sue Neale-May, Jennifer Jenks, Seung-Sin Lee, Kari Nadeau

**Affiliations:** 1 Stanford University, Pediatrics Department, Stanford Medical School, Stanford, USA

## Background

Quantitative measurement of basophil cell surface markers, CD63 and CD203c, by flow cytometry has been developed as useful tool for the diagnosis of IgE mediated allergies. Available methods require large volume of blood and include extra steps such as prior cell purification, priming with IL-3, stopping cell activation and fixing. We have developed a new basophil based test which can be performed with small amount of whole blood directly from skin prick or from small amount of the whole blood where multiple allergens can be tested at the same time for high-throughput testing.

## Materials and Methods

Patients who have been diagnosed with the peanut allergies were examined for their CD203c and CD63 inducible expression after the in vitro activation. Whole blood, 1-2 drops, was collected by skin prick in to the allergen and anti-coagulant coated 96 well plate. Alternatively 3-5 ml of the whole blood was collected in the EDTA or Heparin coated tubes and 100μl was incubated with multiple allergens in 96 plate for high-throughput testing. After 20 minutes of incubation at 37°C, the whole blood was stained with fluorochrome conjugated antibodies and lysed with ammonium chloride buffer. Basophil characterization was done by gating on CCR3High and SSClow cells. Eosinophils were excluded on the basis of granularity and T cells were excluded on the basis of low expression of CCR3. The CD203c or CD63 expression was analyzed before and after stimulation.

## Results

The CD203c expression on basophils, as gated on the CCR3High and SSClow cells, showed shift of the mean fluorescence intensity (MFI) before and after activation with allergens. Control allergens did not show any change in the MFI before and after the incubation, which suggested allergen specific CD203c induction on basophil cell surface. This shift in the CD203c cell surface expression was consistent with the results obtained with other basophil stimulation tests used in allergy diagnosis. However our new method is fast and efficient in performing allergy diagnosis which does not require extra steps used by other protocols for example use of two or more basophil markers, basohil priming and fixing of cells.

